# An Evaluation of Electroacupuncture at the Weizhong Acupoint (BL-40) as a Means of Relieving Pain Induced by Extracorporeal Shock Wave Lithotripsy

**DOI:** 10.1155/2014/592319

**Published:** 2014-07-23

**Authors:** Wei-Ta Chen, Fang-Chia Chang, Yi-Hung Chen, Jaung-Geng Lin

**Affiliations:** ^1^Department of Chinese Medicine, Taiwan Landseed Hospital, No. 77, Kwang-Tai Road, Ping-jen City, Tao-Yuan County 32405, Taiwan; ^2^Department of Veterinary Medicine, National Taiwan University, No. 1, Section 4, Roosevelt Road, Taipei 10617, Taiwan; ^3^Graduate Institute of Acupuncture Science, China Medical University, No. 91 Hsueh-Shih Road, Taichung 40402, Taiwan; ^4^School of Chinese Medicine, China Medical University, No. 91 Hsueh-Shih Road, Taichung 40402, Taiwan

## Abstract

*Background*. Extracorporeal shock wave lithotripsy (ESWL) is the preferred option for urolithiasis treatment. However, intensities of pain may be induced and the sedative anesthetic or analgesics were usually needed. The aim of this study was to develop an improved acupuncture-assisted anesthesia approach in pain relief. *Methods*. We conducted a single-blind, randomized controlled study in China Medical University Hospital. Patients treated by ESWL due to upper urolithiasis were randomly divided into control group, sham-EA group, and 100 Hz EA group. The high frequency electroacupuncture (EA) was applied at the Weizhong acupoint (100 Hz EA group) for 20 minutes prior to the ESWL. In the sham-EA group, the same procedures were performed as those of 100 Hz EA group but no electric current was given to stimulate the acupoints. In the control group, no action was taken before operation. The information including the numbers and dosage of analgesic requirements, pain score, vital signs, and the satisfaction of procedure was collected. *Results*. A total of 74 subjects were recruited and we found that the interval to the first request analgesic, the number/total dosage of additional analgesic, recovery time from anesthesia, and the satisfaction were all better in both the 100 Hz EA and the sham-EA group. The 100 Hz EA also showed better relief of painful sensations by delaying the onset of pain. *Conclusions*. The 100 Hz EA and the sham-EA can effectively relieve pain due to ESWL as well as reducing the dosage of opium analgesic used.

## 1. Introduction

Urolithiasis is one of the most commonly diagnosed diseases of the urinary system, and the prevalence rate in Taiwan is as high as 9%. This rate is showing a very significant increase with time [1]. The causes of urolithiasis can be quite varied and complicated; normally, it is classified based on either an external or an internal origin. External origins refer to environmental factors such as geographic distribution, climate, season, water uptake, diet, and occupation. Internal origins refer to congenital biochemical factors (physiological characters) or anatomic characteristics such as heredity, age, and gender [1, 2]. At present, extracorporeal shock wave lithotripsy (ESWL) is the preferred option for the treatment of upper urolithiasis. The strength of the electrical shock wave when the development of lithotripsy was at an early stage tended to be stronger and this caused perceptible pain for patients; as a consequence, general anesthesia, spinal cord, or epidural anesthesia was often performed [3, 4]. In recent years, the development of new lithotripter models and a trend towards outpatient lithotripsy has resulted in lithotripsy moving toward being a painless treatment. This is because patients tend to benefit from a better and faster postoperational recovery in such circumstances. This trend has also been helped by better and more diversified sedative and anesthetic techniques [4]. It is anticipated that the improvements in anesthetic technology and the enhancement of the lithotripsy apparatus will substantially reduce the amount of sedative anesthetic and analgesic administered and this, in turn, will decrease the side effects that arise from these drugs. This will lead to a reduction in patient recovery time for both outpatients and inpatients as well as shorter hospital stays for the patients. Acupuncture is an important treatment approach in the Chinese traditional medicine, and acupuncture anesthesia has long been critically acclaimed by medical researchers [5–13]. This study was aimed at acquiring an in-depth understanding of the functionality of 100 Hz EA as a pain relief method among patients undergoing ESWL.

## 2. Methods

### 2.1. Patient Selection

After obtaining the consent of the institutional review board, seventy-four patients were recruited from the China Medical University Hospital. These patients suffered from upper urolithiasis and had a confirmed diagnosis from the urologist indicating treatment by ESWL. These patients were viewed as types ASA-I and ASA-II, which classify patients as generally in good health status or with only minor systemic disorders without functional abnormalities. The patient indications that were considered to be appropriate for ESWL included symptoms of hematuria, pain, hydronephrosis, or other urinary infections. The criteria for ESWL fitted the following profile: the size and width of the calculi in the ureter were equal to or smaller than 1.0 cm and/or the size of the calculi in ureter was equal to or smaller than 0.5 cm with obvious obstruction as observed by IVP examination.

### 2.2. Experiment Grouping

Patients were divided by randomization into three groups, namely, the control group, the sham-EA group, and the 100 Hz EA group (each group was composed of 24-25 people). In the control group, no action was taken before operation. After lying prone to rest for 20 minutes, the patients underwent extracorporeal shock wave lithotripsy via a Compact Delta prototype produced by the Dornier Company, Germany. For the sham-EA group, before the operation, the patients lying prone on the treatment bed were subject to 75% ethanol sterilization of the Weizhong acupoint and a nonmeridian, nonacupoint target site 3 cm away from the Weizhong site. Two 30 gauge stainless steel acupuncture needles were inserted at the Weizhong acupoint and the nonacupoint on the leg of the affected side of urolithiasis. For both acupuncture sites, no “de-qi” was induced. Both acupuncture sites were set up with the electrostimulator (Trio 300 electrostimulator, 3-3-3 Toyotama-Minami, Nerima, Tokyo, Japan) connected to the patients (negative pole attached to the Weizhong site and the positive pole attached to the nonmeridian/collateral nonacupoint location). However, although the function key of the electrostimulator was switched on, no electric current was produced to stimulate the acupoint. After 20 minutes of sham-EA, the patients were subjected to ESWL. With the 100 Hz EA group, before the operation, the patients lay prone on the treatment bed and were subject to 75% ethanol sterilization of the Weizhong acupoint and a nonmeridian, nonacupoint target site 3 cm away from the Weizhong site. Two 30 gauge stainless steel acupuncture needles were inserted at the Weizhong acupoint and the nonacupoint, which was 3 cm away from the Weizhong, on the leg of the affected side of urolithiasis. Only the Weizhong acupoint was stimulated and “qi” was induced (the patient reported the sensation of “de-qi”). The other point had no “de-qi”. Both acupuncture sites were set up with the electrostimulator (Trio 300 electrostimulator, 3-3-3 Toyotama-Minami, Nerima, Tokyo, Japan) connected to the patients (negative pole attached to the Weizhong site and the positive pole attached to the nonmeridian/collateral nonacupoint location) to form a pair of electric circuits using 100 Hz frequency. The pulse wave (sphygmogram) was set with width of 100 *μ*s and an appropriate amperage of 1~2 mA at a level where the patient was aware of sensation and the muscles were observed to pulsate slightly. The patients were stimulated for 20 minutes and then underwent ESWL.

### 2.3. Extracorporeal Shock Wave Lithotripsy Protocol

After the preoperational X-ray images of patients were read to confirm the location and size of the calculi, physiological monitors (EKG, BP, and PaO_2_) were connected to patients, and dormicum (0.04 mg/kg) was injected through an intravenous drip and lithotripsy initiated. When a patient started to raise a hand or move in a manner such that it interfered with the lithotripsy operation, a pain score was obtained from the patient. When the pain score exceeded 3 points, this immediately prompted one administration of alfentanil (3 *μ*g/kg). The duration of the lithotripsy operations was between 50 and 60 minutes and the number of shock waves generated was about 3000. During the process of lithotripsy, the strength of shock wave gradually increased. The strength of shock wave was programmed at 11 KV for shots 1–100, at 12 KV for shots 101–500, and at 13 KV for shots 500 during the remaining shock waves.

### 2.4. Recorded Items

The following information was recorded during operation: (1) patients' requests for analgesia indicated by raising their hand during the operation as the time of first raising the hand, the number of times the hand was raised for analgesia, the pain visual analogue scale (VAS) values indicated by the patients, and the dosage opium derivative analgesic given; (2) the vital signs such as blood pressure, heart rate, and arterial oxygen partial pressure; and (3) any relevant side effects induced by sedatives and opium derivative analgesic such as nausea, vomit, dizziness, and itching. In addition, the following information was also recorded in the recovery room: (1) any side effects after operation; (2) the recovery time after anesthesia; and (3) a grading on the level of satisfaction with respect to pain control during the operation.

### 2.5. Statistical Analysis

SAS8.01 computer software was used to calculate the statistical analysis. The distribution of age, height, weight, size of calculi, strength of lithotripsy shock waves, number of shock waves generated, operational time of the lithotripsy, number of times the patient's hands were raised, the dosage of the drugs, the recovery time after anesthesia, the VAS values, and so forth all exhibited a median value (25%–75%) by the Kruskal-Wallis test. A Scheffe test was performed after trial to confirm any statistically significant differences (*P* < 0.05) between the three groups and when the 1st pain score was collected. Fisher's least significant difference method was used after analysis to confirm any statistically significant differences (*P* < 0.05). The time when the patients first raised their hands was analyzed using Kaplan-Meier method for survival analysis in order to predict the survival equation of the time of first raising a hand and the log-rank test was carried out to verify any statistically significant difference (*P* < 0.05) between the groups. Gender, ASA, location of calculi, side effects of the analgesic, patients satisfaction regarding pain controllability, and relevant side effects caused by the opium derivative analgesic were assessed by the *χ* ^2^ test or Fisher's exact test; specifically, when less than 20% of columns had expected values smaller than 5, the *χ* ^2^ test was used and, conversely, when more than 20% of columns had values smaller than 5, Fisher's exact test was used. The raising of a hand during the operation to boost drug dosage was evaluated by *χ* ^2^ test as long as a significant difference was picked up between groups and then a logic regression approach was adopted to explore after analysis the groups having significant variation.

## 3. Results

### 3.1. Demographic Data Analysis of Each Group

When the demographic data of the seventy-four patients undergoing treatment with ESWL were analyzed, it was found that gender, ASA body type, age, height, and weight factors did not show any statistically significant variation (Table 1).

### 3.2. Analysis of the Location and Size of the Calculi

The location of the upper urolithiasis, whether it was on the left and right sides, at the ureteral pelvis junction (UPJ), or at upper part of the ureter together with the size of calculi, did not show any statistically significant variation (Table 2).

### 3.3. Analysis of the Strength of the Lithotripsy Shock Waves, the Shock Wave Count, and the Duration of Lithotripsy Operation

The factors associated with the lithotripsy operation for upper urolithiasis did not demonstrate any statistically significant variation (Table 3).

### 3.4. Drug Related Data Analysis during Operation (Table 4)

1. Number of patients who have not raised their hands for analgesic during operation: there were more patients within the 100 Hz EA group than the control group who did not raise a hand and the difference was statistically significant (*P* < 0.05).
2. The time until the first raising of a hand for analgesia during operation: compared to the control group, the 100 Hz EA group and sham-EA group took 35 minutes more and 21 minutes more, respectively, to raise their hands and this was statistically significant (*P* < 0.001).
3. The number of hand raising events for analgesia during operation among the 100 Hz EA group and the sham-EA group was twice and once fewer than the control group and this was statistically significant (*P* < 0.001).
4. The total dosage of administrated anesthetic in each group had no statistical difference.
5. The dosage of analgesic provided to the 100 Hz EA group was 210.00 *μ*g/kg less than that provided to the control group, and this was statistically significant (*P* < 0.01).
6. The total dosage of analgesic requested by raising a hand during the operation among the 100 Hz EA group and the sham-EA group was 462.00 *μ*g/kg and 309.17 *μ*g/kg less than the control group, respectively, and this was statistically significant (*P* < 0.001).
7. When the recovery time after the anesthesia was analyzed, the 100 Hz EA group and the sham-EA group required 10-minute less recovery time than the control group and this was a statistically significant variation (*P* < 0.001).

### 3.5. Time Survival Analysis for First Hand Raising for More Analgesic (Figure 1)

1. In total, 25% of the patients had requested first analgesic at 3.25 minutes in the control group, at 10 minutes in the sham-EA group, and at 18.25 minutes in the 100 Hz EA group.
2. In total, 40% of the patients had requested first analgesic at 7 minutes in the control group, at 24 minutes in the sham-EA group, and at 25 minutes in the 100 Hz EA group.
3. At the end of the study, 20% of the patients had not raised their hands for analgesic in control group, 45.83% had not raised their hands for analgesic in sham-EA group, and 56% had not raised their hands for analgesic in 100 Hz EA group.

### 3.6. Pain Score Analysis during Operation (Table 5)

1. The pain score for 100 Hz EA group at first hand raising for analgesic was 3 points lower than that of the control group and this was statistically significant (*P* < 0.05).
2. The highest pain score during operation for the 100 Hz EA group was 4 points less than that of the control group and this was statistically significant (*P* < 0.01).
3. The pain scores for the 100 Hz EA group and the sham-EA group after analgesic was administered were 1 point lower than the control group and this was statistically significant (*P* < 0.001).

### 3.7. Analgesic Side Effects Analysis (Table 6)

Each group was analyzed and compared based on the side effects of the analgesia, but there was no statistically significant variation.

### 3.8. Pain Controllability Satisfaction Analysis (Table 7)

1. Within the 100 Hz EA group, 80% of the patients were very satisfied, 20% were satisfied, and 0% were slightly satisfied, unsatisfied, or completely unsatisfied.
2. Within the sham-EA group, 54% of the patients were very satisfied, 46% were satisfied, and 0% were slightly satisfied, unsatisfied, or completely unsatisfied.
3. Within the control group, 16% of the patients were very satisfied, 32% were satisfied, 52% were slightly satisfied, and 0% were unsatisfied or completely unsatisfied.

Thus both the 100 Hz EA group and the sham-EA group had a high percentage of patients showing a high level of satisfaction compared to the control and this was statistically significant.

## 4. Discussion

In acupuncture theory, the bladder meridian separated into two submeridians from the posterior neck and passed through the lumbar region. Then the two submeridians had been integrated at the Weizhong acupoint behind the knee joints. Hence, we can use Weizhong acupoint as an important acupoint in treating back and waist pain problems. Based on this, all diseases originating from waist area can be treated [14]. Based on traditional Chinese medicine, upper urolithiasis is located at this waist area, where the bladder meridian enters the abdominal cavity connecting the kidneys to the bladder. Furthermore, peripheral and organ pain felt during ESWL are also inside this waist area. Based on this, we targeted the Weizhong acupoint as the priority site for acupuncture before proceeding with the ESWL. The Weizhong acupoint is located behind knee joints, passes through the midpoint of popliteal striated muscle, and resides in between the biceps femoris tendon and semitendinosus muscle tendon [15].

Current ESWL related acupuncture studies have used different acupoint selections and most studies have adopted simultaneous stimulation at multiple sets of acupoints [16–18]. Sun et al. implemented electroacupuncture to treat patients undergoing ESWL and showed that 85% of patients did not require analgesic drugs to relieve pain [17]. Similarly, the results of Wang et al. showed that 85% of electroacupuncture and 70% of manual acupuncture patients did not require analgesic drugs [16]. Chan et al. also reported similar results [19]. This suggests that acupuncture and electroacupuncture are both methods of effective analgesia for patients undergoing ESWL; this agrees with the results of this study. Specifically, this study abides by the Chinese traditional medical theory and obeys the idea that “diseases are managed where meridians pass by,” “meridians flow by where the principle target treatment site resides,” and “if the disease attacks the head, the foot should be treated; if the disease attacks waist, the popliteal area should be treated.” In this study, based on the above approach, the Weizhong acupoint is justified as the site of choice as it is placed on the bladder meridian.

The best-known mechanism of acupuncture analgesia is via endogenous opiates and their receptors [20]. Different kinds of endogenous opiates, such as *β*-endorphin, enkephalin, endomorphin, and dynorphin, reportedly act as frequency-dependent factors in EA. EA of low frequency (2 Hz) accelerated the release of *β*-endorphin and enkephalin in the CNS whereas EA of high frequency (100 Hz) accelerated the release of dynorphin [21]. However, in our previous study, we found that high frequency EA is more effective than low frequency EA in clinical evaluation. We examined the effects of preoperative EA at classical bilateral acupuncture points (Zusanli, also known as ST-36) on postoperative pain [11]. Patients undergoing lower abdominal surgery were randomly assigned to four treatment regimens: control; sham-EA (needle insertion without electrical stimulation); low-EA (2 Hz of electrical stimulation); and high-EA (100 Hz of electrical stimulation). Postoperative pain was evaluated by recording the total amount of morphine required by PCA. We found that, during the first 24 h, the total amount of morphine required was decreased by 21, 43, and 61% in the sham-, low-, and high-EA groups, respectively. Therefore, this study only included three groups, namely, a control group, a sham-EA group, and a 100 Hz EA group. No low frequency (2 Hz) electroacupuncture group was included because many studies already support the efficacy of high frequency (100 Hz) electroacupuncture compared to the low frequency (2 Hz) electroacupuncture.

In the study, no significant difference in analgesia between the 100 Hz EA group and the sham-EA group was found. This is presumed to be due to a number of factors. Firstly, the type of pain and its intensity as induced by the ESWL procedure are different from the pain endured after an anesthetic has worn off during operations at other anatomical sites, such as the thoracic cavity and abdominal cavity. It is well recognized that the most painful sensations among all types of operations are the ones that involve postoperational pain associated with the thoracic cavity or upper abdominal area. This is followed by the lower abdominal operation and peripheral operations are generally the least painful. The primary origin of the pain generated in lithotripsy is due to the shock wave passing through the skin, which induces peripheral pain and this is followed by organ pain induced by effects of the focused shock wave on the area around the kidney where there are nerves distributed in the capsule. Thus, in general, the pain intensity of ESWL is lower. Even, the pain intensity of the ureteral pelvis junction (UPJ) and the upper part of the ureter is lower than the area around the kidney during ESWL operation. Secondly, the structure of the Weizhong acupoint is complicated, and its characteristics are different from the ones we have used previously [11, 22–24]. These acupoints are located close to muscle structures, such as the Zusanli (足三里), the Sanyinjiao (三陰交), and the Yanglingquan (陽陵泉). The distribution of nerves and blood vessels is more condensed and complicated at Weizhong acupoint and therefore any manipulation of the Weizhong acupoint demands delicate needle movements. This requirement might compromise qi induction. Thirdly, sham-EA can alleviate pain and this had been demonstrated previously [11]. This is because, during the quite accurate inserted at Weizhong accupoint process by the practitioner, the quivering of needling might stimulate the sham-EA group causing qi induction. Fourthly, the dosage level for the acupuncture is low with this study only selecting one acupoint, the Weizhong; this differs from our previous studies [11, 22–24] and other ESWL related acupuncture studies [16–18, 25], where multiple acupoints have been used. Consequently, the dosage of acupuncture applied in this study is relatively lower. Fifthly, the effect of the analgesic is optimal and the action of relieving pain is quick. Furthermore, alfentanil is a better analgesic and has a more superior recovery time than other commonly used operation room drugs such as fentanyl. Sixthly, due to the characteristics of Weizhong acupoint, electroacupuncture induced electrical stimuli are not evident to the patient. Finally, the number of participating patients in this study is still insufficient. All of the above factors lead to the conclusion that the analgesia effects of the 100 Hz EA group and the sham-EA group do not show statistically significant variation.

To the best of knowledge, this is the first study to report that 100 Hz EA and sham-EA can effectively relieve pain due to ESWL as well as reducing the dosage of opium analgesic used. However, it is still unclear how the placebo effect of EA contributes to analgesic effect in the present study since sham-EA was also effective. Future studies with different control group design are needed. We have previously reviewed the control group design in acupuncture randomized controlled trials (RCTs) and proposed four different strategies for designing control groups: (1) absence of acupuncture needle insertion, (2) different location of inserted acupuncture needles, (3) different depth of insertion, and (4) the use of assistant tools [26]. These four strategies can be considered when designing the next study.

The number of lithotripsy shock waves is usually programmed in accordance with the patient's heart rate and if the patient exhibits abnormal heart beats, medication is often administered to control the patient's heart rate within a normal range. As a result, differences in the duration of the lithotripsy period between the groups were quite minor. The Weizhong acupoint is capable of recuperating the urinary bladder from qi transformation and modulating the functioning of lower-jiao qi activity (下焦氣機) as well as producing a quick response pain relief through fluid circulation of qi. It also helps to facilitate hemocirculation to remove stasis and move qi in order to quickly alleviate smooth muscle spasms. This feature will help relieve the operational pain and move a foreign object like the calculi downwards for excretion [27]. Further studies are warranted to confirm whether targeting Weizhong acupoint for acupuncture may reduce the operation time for lithotripsy.

Study of the opiate related side effects shows that the occurrence rates for each group were generally low and it is presumed that the rationale for the low level of alfentanil related side effects is, firstly, that the originally single dosage of alfentanil is already low (3 *μ*g/kg) and, secondly, that the superior effectiveness of analgesia with electroacupuncture makes the dosage of alfentanil needed in the electroacupuncture group lower, which, in turn, leads to fewer side effects.

Recovery time from anesthesia is evaluated by the anesthesiologist and the standards of recovery for anesthesia are as follows: consciousness, no dizziness when moving from a sitting position to upright standing, ability to walk around voluntarily without assistance [7], and a lack of discomfort or anguish even upon standing up [28]. Resim et al. showed that electroacupuncture can effectively reduce the side effects of ESWL, which benefits early recovery.

The study further supports the analgesia effect of electroacupuncture and it is clear that, despite the use of different acupoints selected in various anatomical areas using a diversity of selection principles, they all are able to fulfill the objective of pain relief. This study also implies that further study is warranted to explore which operations should be combined with which appropriate acupuncture point(s).

## Figures and Tables

**Figure 1:**
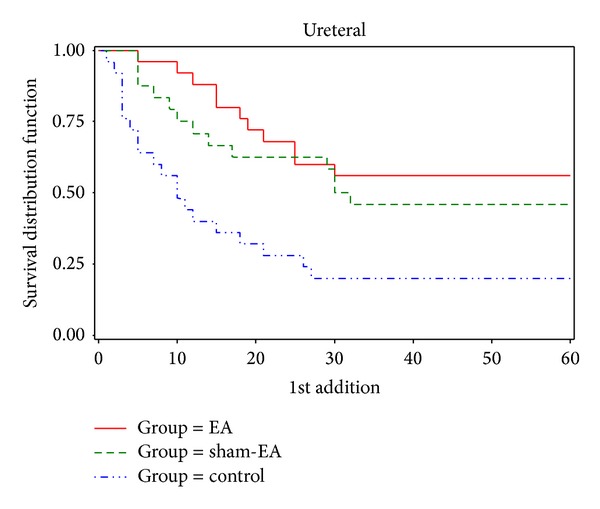
Time survival analysis for the first hands up to request for analgesic between different upper urolithiasis groups.

**Table 1:** Demographic data analysis.

	U-I control	U-II sham-EA	U-III 100 Hz EA	*P* value
Number of people/group	25	24	25	
Gender				
Men: *n* (%)	20 (80%)	21 (88%)	18 (72%)	0.4021
Female: *n* (%)	5 (20%)	3 (12%)	7 (28%)	
ASA				
ASA-I: *n* (%)	16 (64%)	18 (75%)	20 (80%)	0.4282
ASA-II: *n* (%)	9 (36%)	6 (25%)	5 (20%)	
Age (yr)	48.00 (40.00–53.00)	42.50 (34.50–49.00)	44.00 (36.00–48.00)	0.2327
BH (cm)	165.00 (162.00–171.60)	166.50 (162.80–172.50)	166.00 (162.00–172.00)	0.9230
BW (kg)	74.00 (65.00–80.00)	70.50 (63.75–80.00)	63.10 (61.00–76.70)	0.2539

Median (25%–75%).

**Table 2:** Location and size of upper urolithiasis analysis.

Number of people with calculi at the specific location and size of calculi (%)	U-I control (*n* = 25)	U-II sham-EA (*n* = 24)	U-III 100 Hz EA (*n* = 25)	*P* value
Position				
L side stone	17 (68%)	14 (58%)	13 (52%)	0.5101
R side stone	8 (32%)	10 (42%)	12 (48%)	
Location				
UPJ	4 (16%)	1 (4%)	3 (12%)	0.5154
Upper	21 (84%)	23 (96%)	22 (88%)	
Middle	0 (0%)	0 (0%)	0 (0%)	
Low	0 (0%)	0 (0%)	0 (0%)	
Stone size (mm^2^)	45.00 (36.00–70.00)	45.00 (32.00–81.00)	54.00 (35.00–77.00)	0.9725

Median (25%–75%).

**Table 3:** Strength of lithotripsy shock wave for upper urolithiasis, counts of shock wave generated, and duration of lithotripsy operation.

ESWL data	U-I control (*n* = 25)	U-II sham-EA (*n* = 24)	U-III 100 Hz EA (*n* = 25)	*P* value
Shock wave intensity (KV)	13.00 (13.00-13.00)	13.00 (13.00-13.00)	13.00 (13.00-13.00)	0.8724
Total shock wave delivered	3000 (3000-3000)	3000 (3000-3000)	3000 (3000-3000)	0.3997
Duration of ESWL (min)	55.00 (45.00–60.00)	52.50 (47.50–55.00)	50.00 (45.00–55.00)	0.3422

Median (25%–75%).

**Table 4:** Different upper urolithiasis group and drug related data analysis.

	U-I control (*n* = 25)	U-II sham-EA (*n* = 24)	U-III 100 Hz EA (*n* = 25)	*P* value	Multiple comparison
Number of people who raise hands for analgesic (%)					
No	5 (20%)	11 (46%)	14 (56%)	0.0282∗	I_III
Yes	20 (80%)	13 (54%)	11 (44%)		
Time of first hands up (minutes)	10.00 (4.00–26.00)	31.00 (11.00–>50.00)	>45.00 (19.00–>45.00)	0.0003∗∗∗	I > II, I > III
Counts of hands up for more analgesic	2.00 (1.00–4.00)	1.00 (0.00–1.50)	0.00 (0.00–1.00)	0.0002∗∗∗	I > II, I > III
Total dormicum (mg/kg)	2.96 (2.52–3.20)	2.80 (2.68–3.15)	2.60 (2.44–3.02)	0.2847	
Alfentanil (ug/kg)	210.00 (170.00–228.00)	162.00 (0.00–210.00)	0.00 (0.00–189.00)	0.0096∗∗	I > III
Total alfentanil (ug/kg)	462.00 (228.00–756.00)	152.83 (0.00–236)	0.00 (0.00–220.00)	<0.0001∗∗∗	I > II, I > III
Recovery time (min)	15.00 (10.00–20.00)	5.00 (5.00–15.00)	5.00 (2.00–6.00)	<0.0001∗∗∗	I > II, I > III

Median (25%–75%).

∗ *P* < 0.05, ∗∗*P* < 0.01, and ∗∗∗*P* < 0.001.

**Table 5:** Pain scores for different upper urolithiasis groups.

VAS	U-I control (*n* = 25)	U-II sham-EA (*n* = 24)	U-III 100 Hz EA (*n* = 25)	*P* value	Multiple comparison
1st pain score	3.00 (3.00–4.00)	3.00 (0.00–3.00)	0.00 (0.00–3.00)	0.0439∗	I > III
Max pain score	4.00 (3.00–5.00)	3.00 (0.00–3.50)	0.00 (0.00–3.00)	0.009∗∗	I > III
Controlled pain score	1.00 (0.00–2.00)	0.00 (0.00-0.00)	0.00 (0.00-0.00)	<0.001∗∗∗	I > II, I > III

Median (25%–75%).

∗ *P* < 0.05, ∗∗*P* < 0.01, and ∗∗∗*P* < 0.001.

**Table 6:** Analgesic side effects for different upper urolithiasis groups.

Side effect of analgesic Number of people (%)	U-I control (*n* = 25)	U-II sham-EA (*n* = 24)	U-III 100 Hz EA (*n* = 25)	*P* value
Do not feel dizzy at all after operation (score 0)	17 (68%)	22 (92%)	21 (84%)	0.1009
Feel slightly dizzy after operation (score 1)	7 (28%)	1 (4%)	4 (16%)	
Feel dizzy and walk wobbly after operation (score 2)	1 (4%)	1 (4%)	0 (0%)	
Feel very dizzy, walk wobbly, and nauseate after operation (score 3)	0 (0%)	0 (0%)	0 (0%)	
Feel very dizzy, walk wobbly, and vomit after operation (score 4)	0 (0%)	0 (0%)	0 (0%)	

**Table 7:** Pain controllability satisfaction analysis for patients in different upper urolithiasis groups.

Patients' satisfaction level for pain controllability Number of people (%)	U-I control (*n* = 25)	U-II sham-EA (*n* = 24)	U-III 100 Hz EA (*n* = 25)	*P* value
4 = very satisfied	4 (16%)	13 (54%)	20 (80%)	0.00∗∗∗
3 = satisfied	8 (32%)	11 (46%)	5 (20%)	
2 = slightly satisfied	13 (52%)	0 (0%)	0 (0%)	
1 = unsatisfied	0 (0%)	0 (0%)	0 (0%)	
0 = very unsatisfied	0 (0%)	0 (0%)	0 (0%)	

***P < 0.001.

## References

[1] Dong YP, Sun GH, Yu DH (1996). Epidemiology and root case of urolithiasis. *The Most Updated Development of Urolithiasis Treatment*.

[2] Lee Y, Chang H, Lee Y, Chen M, Chang H (1991). Epidemiology of urolithiasis. *Urolithiasis*.

[3] Schockenhoff B, Daub D, Stadermann D, Rubben H (1987). Opioid analgesia in extracorporeal shock wave lithotripsy: fentanyl versus alfentanil. *European Urology*.

[4] Ling YS, Liu CH, Lin JR (1993). Comparison of intravenous alfentanil, fentanyl and epidural lidocaine application on extracorporeal shock wave lithotripsy. *Acta Anaesthesiologica Sinica*.

[5] Wedenberg K, Moen B, Norling A (2000). A prospective randomized study comparing acupuncture with physiotherapy for low-back and pelvic pain in pregnancy. *Acta Obstetricia et Gynecologica Scandinavica*.

[6] Highfield ES, Kaptchuk TJ, Ott MJ, Barnes L, Kemper KJ (2003). Availability of acupuncture in the hospitals of a major academic medical center: a pilot study. *Complementary Therapies in Medicine*.

[7] Nakanishi O, Jingxue R, Amano Y, Tsuru N, Nishi M (1995). Comparison of the effects of acupuncture and nitrous-oxide sedation on tactile and pain sensations in the right lower lip region. *Anesthesia Progress*.

[8] Luo H (2000). Clinical case report: breast lumpectomy with acupuncture anesthesia. *Clinical Acupuncture and Oriental Medicine*.

[9] Kalish LA, Buczynski B, Connell P (2004). Stop Hypertension with the Acupuncture Research Program (SHARP): clinical trial design and screening results. *Controlled Clinical Trials*.

[10] Koo ST, Park YI, Lim KS, Chung K, Chung JM (2002). Acupuncture analgesia in a new rat model of ankle sprain pain. *Pain*.

[11] Lin JG, Lo MW, Wen YR, Hsieh C, Tsai S, Sun W (2002). The effect of high and low frequency electroacupuncture in pain after lower abdominal surgery. *Pain*.

[12] Christensen PA, Rotne M, Vedelsdal R, Jensen RH, Jacobsen K, Husted C (1993). Electroacupuncture in anaesthesia for hysterectomy. *British Journal of Anaesthesia*.

[13] Lu DP, Lu GP (1993). Acupuncture anesthesia/analgesia for pain and anxiety control in dental practice. Part 2: Techniques for clinical applications. *Compendium*.

[14] Lee YR, Chang YF (2001). Acupuncture treatment on Weizhong point for lumber facture type of waist pain. *Chinese Journal of Integrated Traditional and Western Medicine*.

[15] (1996). *Standard Acupuncture Points illustration*.

[16] Wang SC, Chang SY, Feng SP (1994). Initial observation of alleviation effects induced by acupuncture to relieve pain from extracorporeal shock wave lithotripsy (ESWL). *Chinese Journal of Anesthesiology*.

[17] Sun LH, Wang SC, Feng SP (1994). Application of electroacupuncture anesthetic on extracorporeal shock wave lithotripsy (ESWL). *Journal of Clinical Acupuncture & Moxibustion*.

[18] Rogenhofer S, Wimmer K, Blana A, Roessler W, Wieland WF, Filbeck T (2004). Acupuncture for pain in extracorporeal shockwave lithotripsy. *Journal of Endourology*.

[19] Chang H, Chen Y, Kao C (2000). Acupuncture anesthetic application for extracorporeal shock wave lithotripsy on elders. *Journal of Clinical Acupuncture & Moxibustion*.

[20] Han J (2011). Acupuncture analgesia: areas of consensus and controversy. *Pain*.

[21] Han JS (2003). Acupuncture: Neuropeptide release produced by electrical stimulation of different frequencies. *Trends in Neurosciences*.

[22] Wei IP (2001). An assessment on the effect of pain relieving after cesarean section operation obtained by applying electroacupuncture of different frequencies on the point of Sanyinchiao (SP-6). *Chinese Medical Science*.

[23] Chen YC, Liu HJ, Lin JG (2005). Assessment on the effect of postoperative acupuncture stimulation to pain relief after total knee replacement. *Taiwan Journal of Chinese Medicine*.

[24] Wu HC (1999). Effects of acupuncture on post-cesarean-section pain. *Chinese Medical Science*.

[25] Lee W, Yang C (1995). Study on analgesia efficacy by auricular needling anesthetic for extracorporeal shock wave lithotripsy. *Chinese Medical Research Journal*.

[26] Lin JG, Chen CH, Huang YC, Chen YH (2012). How to design the control group in randomized controlled trials of acupuncture?. *Evidence-Based Complementary and Alternative Medicine*.

[27] Shan S, Chang R (2004). 34 examples of acupuncturing Weizhong acupoint to treat kidney angina. *Journal of Clinical Acupuncture & Moxibustion*.

[28] Yang C, Cherng C, Wong C, Ho S (2002). Effects of intravenous ketorolac and fentanyl combined with midazolam on analgesia and side effects during extracorporeal shock wave lithotripsy. *Acta Anaesthesiologica Sinica*.

